# Reverse Engineering of Oxygen Transport in the Lung: Adaptation to Changing Demands and Resources through Space-Filling Networks

**DOI:** 10.1371/journal.pcbi.1000902

**Published:** 2010-08-26

**Authors:** Chen Hou, Stefan Gheorghiu, Virginia H. Huxley, Peter Pfeifer

**Affiliations:** 1 Department of Systems and Computational Biology, Albert Einstein College of Medicine, Bronx, New York, United States of America; 2 Center for Complexity Studies, Bucharest, Romania; 3 Department of Medical Pharmacology and Physiology, University of Missouri, Columbia, Missouri, United States of America; 4 Department of Physics, University of Missouri, Columbia, Missouri, United States of America; University of Washington, United States of America

## Abstract

The space-filling fractal network in the human lung creates a remarkable distribution system for gas exchange. Landmark studies have illuminated how the fractal network guarantees minimum energy dissipation, slows air down with minimum hardware, maximizes the gas- exchange surface area, and creates respiratory flexibility between rest and exercise. In this paper, we investigate how the fractal architecture affects oxygen transport and exchange under varying physiological conditions, with respect to performance metrics not previously studied.

We present a renormalization treatment of the diffusion-reaction equation which describes how oxygen concentrations drop in the airways as oxygen crosses the alveolar membrane system. The treatment predicts oxygen currents across the lung at different levels of exercise which agree with measured values within a few percent. The results exhibit wide-ranging adaptation to changing process parameters, including maximum oxygen uptake rate at minimum alveolar membrane permeability, the ability to rapidly switch from a low oxygen uptake rate at rest to high rates at exercise, and the ability to maintain a constant oxygen uptake rate in the event of a change in permeability or surface area. We show that alternative, less than space-filling architectures perform sub-optimally and that optimal performance of the space-filling architecture results from a competition between underexploration and overexploration of the surface by oxygen molecules.

## Author Summary

The possibility of predicting oxygen currents in the human lung under varying conditions may give new understanding of the lung's operation, new therapeutic interventions, and new designs for non-biological transport systems. We introduce such a computation which requires only a pocket calculator and agrees with measured currents within a few percent. It treats the network of airways as a fractal surface and exhibits wide-ranging adaptation to changing process parameters, including tolerance to changes in membrane permeability, near-invariance of trans-membrane oxygen pressure at rest and exercise, and transformation of 180,000 gas exchangers into 1,500,000 exchangers from rest to exercise. We show that alternative architectures perform sub-optimally and that the observed performance results from a competition between underexploration and overexploration of the surface by oxygen molecules.

## Introduction

**Figure 1:**
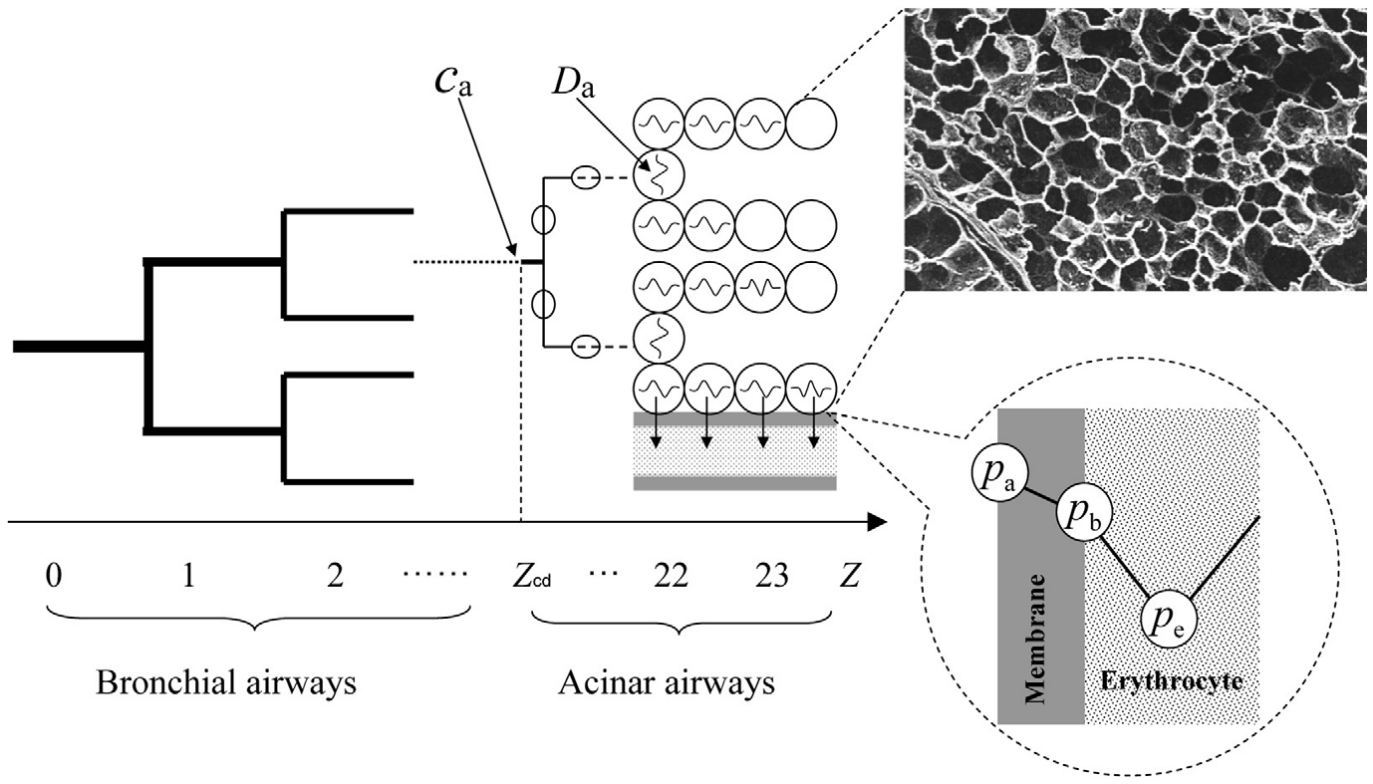
Oxygen transport in the lung, as a function of branching generation *z*. The transition from convection (heavy lines) to diffusion (light lines) occurs at generation *z*_cd_, with 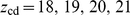 at rest, moderate exercise, heavy exercise, and maximum exercise, respectively (Table 2, see Methods section). The bronchial ducts (*z* = 0–14) do not carry alveoli; the acinar ducts (*z* = 15–23) are lined with alveoli (circles). In the ducts with *z*≥*z*_cd_, O_2_ diffuses through the airways (diffusion coefficient of O_2_ in air, *D*_a_), crosses the alveolar membrane (air-erythrocyte barrier including the alveolar tissue and plasma; hatched; permeability *W*), and binds to an erythrocyte in the capillary.

**Figure 2:**
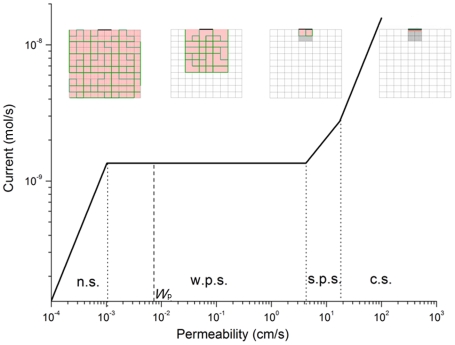
O_2_ current across a 1/8 acinus (gas exchanger at rest). The O_2_ current as a function of permeability, *W*, illustrates decreasing screening from right to left. Far right: diffusion-limited current; far left: reaction-limited current; dashed line: physiological permeability, *W*_p_ = 0.007 cm/s (Table 1). Four insets, in which the square networks are 2-dimensional schematic representations of 1/8 acinus membrane surface, illustrate how accessible regions (from left to right) change as permeability increases. When permeability is small (the inset at far left), the O_2_ diffusion paths of length Λ (green) is long, the oxygen concentration *c*(**x**) = *c*_a_, and the membrane is unscreened (red). As permeability increases, the O_2_ diffusion paths of length are shortened and the red/unscreened areas decrease (the insets from left to right). Symbols n.s., w.p.s., s.p.s., and c.s. denote no screening, weak partial screening, strong partial screening and complete screening, respectively. The calculations from Eqs. 4 and 5 are carried out for a 3-dimension acinus, and the fractal dimension, *D*_f_, is taken to be 3. Except for *W*, which is treated as a variable, the values of all structural and transport parameters in Eqs. 4 and 5 are taken from Table 1 and 2 for a 1/8 acinus (gas exchanger at rest, second column in Table 2).

**Figure 3 pcbi-1000902-g003:**
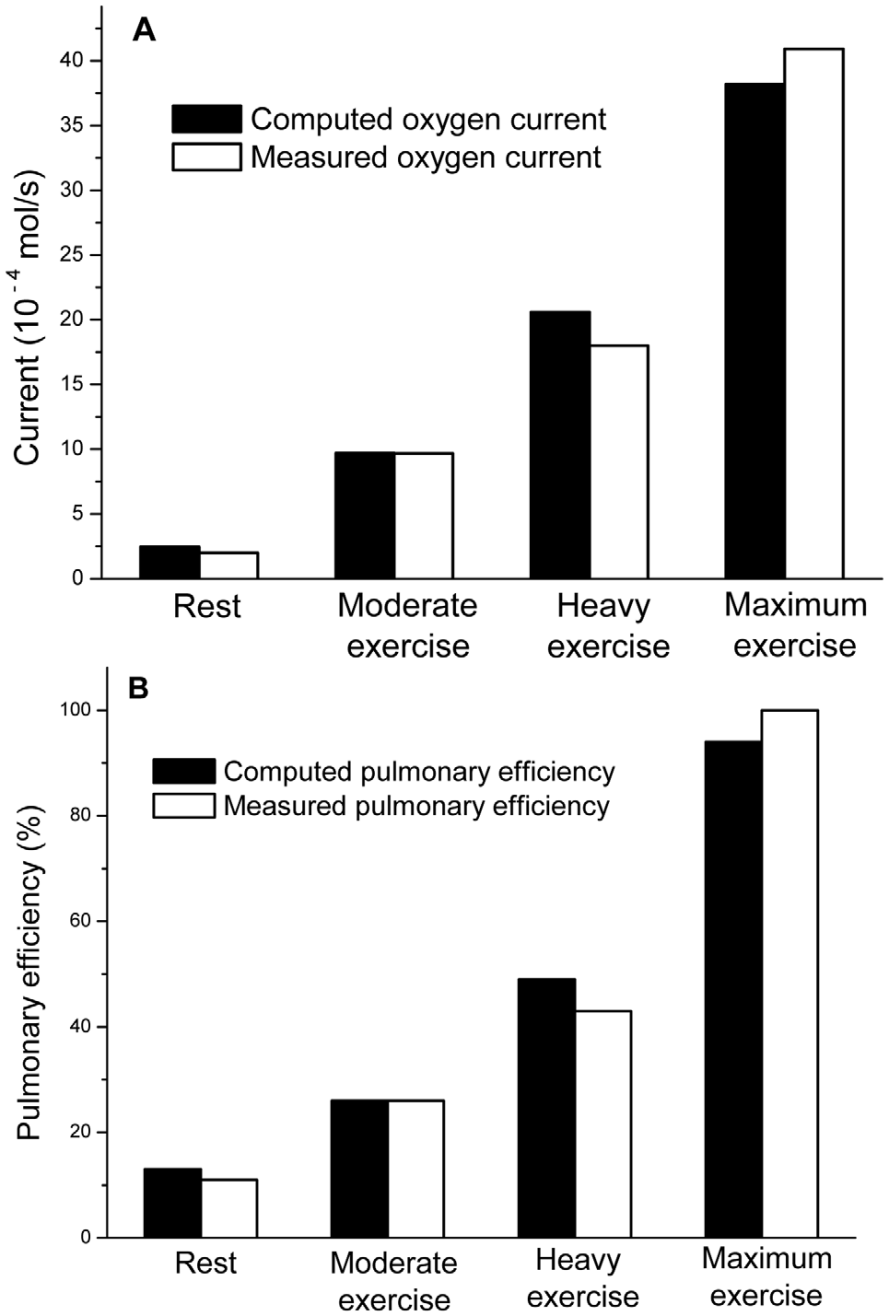
Computed and experimental oxygen currents and pulmonary efficiencies. (A) Computed and experimental O_2_ currents for the whole lung and (B) pulmonary efficiencies, at different levels of exercise (levels of exercise are defined in the Methods section). The currents are computed from Eqs. 4 and 5. The computed pulmonary efficiency is obtained from Eqs. 5 and 7a, and the experimental value of the efficiency is obtained from Eq. 7c. In this figure, we compute the oxygen current with the physiological permeability, so instead of treating *W* as a variable, we take *W* = *W*_p_ (Table 1). Other transport parameters in the equations are listed in Table 1. The structural parameters at different levels of exercise are listed in Table 2 (column 2, 3, 4, and 5). The fractal dimension, *D*_f_, is taken to be 3.

**Figure 4 pcbi-1000902-g004:**
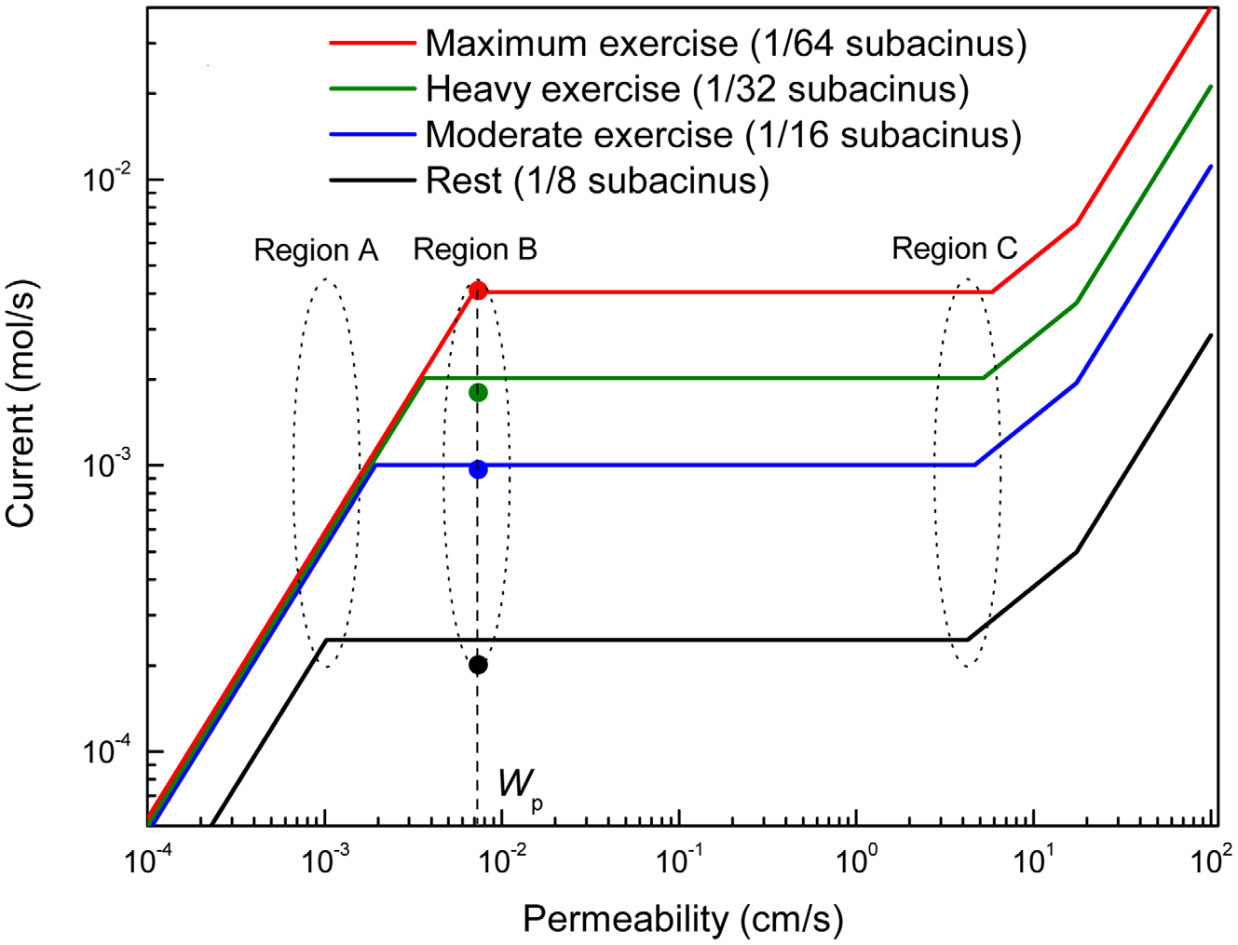
Computed oxygen currents for the lung. Currents for the whole lung are computed for variable *W* and compared with experimental values (dots). Regions A, B, and C show the effects of operating the lung at the physiological value of *W*, *W*_p_, and far way away from *W*_p_ (see text). At moderate, heavy, and maximum exercise, the O_2_ partial pressure difference across the membrane remains essentially constant, 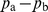 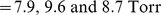 (Table 2). Accordingly, the respective currents are computed replacing the three 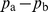 values by their average, 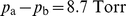, which makes the currents in the no-screening regime, 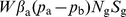, coincide. The currents are computed from Eqs. 4 and 5. Permeability, *W*, is treated as a variable. The transport parameters in the equations are listed in Table 1, and the structural parameters at different levels of exercise are listed in Table 2 (column 2, 3, 4, and 5). The fractal dimension, *D*_f_, is taken to be 3.

On its way from the trachea to blood in the lung, oxygen (O_2_) travels through 14 generations of branching ducts forming the bronchial airways; 9 generations of ducts forming the acinar airways, which end in 300 million alveoli; and across the thin walls separating alveoli and blood capillaries (Fig. 1; [1], [2]). This architecture is a space-filling, fractal network at two levels (Fig. 1; [1]–[4]), each creating a remarkable distribution system: The *space-filling bronchial tree*, in which transport is by convection, guarantees minimum dissipation (pressure-driven flow [5]–[7]), including the 3/4 power law for metabolic rates [8], and slows air down with a minimum number of ducts [4]. In the *space-filling acinar tree*, in which transport occurs primarily by diffusion, the network maximizes the gas-exchange surface area [2], creates respiratory flexibility between rest and exercise [9], and minimizes dissipation, too (diffusion-driven flow [10]). Here we investigate performance metrics (“engineering targets”) of the acinar airways, the fundamental gas exchange system, that have not been studied previously. The metrics will be in terms of diffusive transport, local oxygen currents, and total oxygen currents. They will uncover an unexpected coexistence of new, seemingly mutually exclusive, optimum performance characteristics of the lung. A broad class of structure-function relations for diffusion of molecules to and across biological membranes, under various source and receptor geometries, including associated optimum architectures, has been reviewed in [4].

As air moves through successive branchings of the bronchial and acinar tree, its convective flow velocity decreases as the total cross-section area of the ducts increases. At 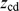 branching generations, the flow velocity equals the diffusion velocity. After this point, although convective flow still exists, the primary transportation mechanism for oxygen becomes diffusion (transition from convection to diffusion; Fig. 1; [9]). The value of 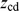 depends on the flow velocity in the trachea, i.e., on the breathing rate. The air at branching generation 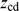 acts as a constant O_2_ source, from which O_2_ diffuses to alveolar membranes downstream, 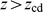, and crosses the membrane into the blood (perfusion). Most of the O_2_ exchange occurs downstream from 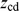, where O_2_ concentration gradients develop as progressively more O_2_ molecules cross the alveolar membrane. The acinus segment 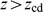 defines a gas exchanger. Each gas exchanger forms a space-filling network, and so does the system of all gas exchangers. The elementary building blocks of the network, small or large, are the alveoli. They manifestly span a 3-dimensional surface (Fig. 1, photographic inset). The drop in O_2_ concentration far from the source is referred to as screening [9], and the oxygen current depends on the degree of screening present in different regions of the gas exchange system. There are two basic mechanisms how screening can vary. One is that the membrane permeability to oxygen, *W* (length per time), may vary, such as under various disease conditions. If *W* is large (small), a molecule crosses after few (many) collisions with the membrane, the O_2_ concentration gradient is large (small), and regions away from the source are screened (unscreened, [9]). The distance the molecule travels along the membrane (acinar ducts) before transfer occurs, is given by 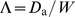 and called exploration length ([4], [11]; also see Methods section), with 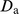 the O_2_ diffusion coefficient in air (area per time). Thus a large permeability generates strong screening and a small exploration length, and vice versa. Under normal conditions, 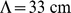 (Table 1). The second mechanism is that the transition from convection to diffusion, 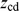, varies with the breathing rate: As ventilation increases under the four levels of exercise considered in this paper—rest and moderate/heavy/maximum exercise (see Methods section for the definition of the levels of exercise)—the flow velocity in the trachea increases and pushes the O_2_ source, 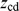, deeper into the acinar tree. The two mechanisms reduce the question of performance of the lung as gas exchange system to the question, what are the relevant length scales downstream of 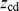 at different levels of exercise? Are they large or small compared to 33 cm? Is the O_2_ current diffusion/access-limited (small Λ) or reaction/transfer-limited (large Λ)?

**Table 1:** Transport parameters to compute O_2_ currents at temperature 310K.

Diffusion coefficient of O_2_ in air, 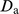 (cm^2^/s) [33]	0.243
Diffusion coefficient of O_2_ in membrane, 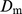 (cm^2^/s) [34]	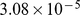
Solubility of O_2_ in air, 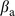 (mol⋅cm^−3^·Torr^−1^)	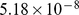
Solubility of O_2_ in membrane, 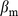 (mol⋅cm^−3^·Torr^−1^) [9]	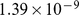
Membrane thickness (including the alveolar tissue and plasma), 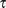 (µm) [12]	1.11
Membrane permeability at physiological conditions, 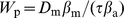 (cm/s)	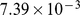

## Methods

### Theoretical Background

The space-filling bronchial airways and space-filling acinar airways are different morphologically (Fig. 1). For the bronchial airways, the duct diameter after z branching generations, *d_z_*, is well-described by Murray's law, *d_z_* = 2^−*z*/3^ *d*_0_, for *z* = 0, …, 14 [1], and generates a tree whose canopy—the collection of all branch tips, has fractal dimension 3 [4]. For the acinar airways, z = 15, …, 23, the diameters remain nearly constant. The ducts and alveoli together span a network that is space-filling, with fractal dimension 3, not just in terms of branch tips, but as a whole, including the ducts (Fig. 11.6 in [1]). In this paper we investigate performance metrics of the acinar airways, through whose surface the gas exchange occurs.

We model the O_2_ transfer from air to blood as a three-step process—diffusion through the acinar airways, diffusion across the membrane, and diffusion and binding to red blood cells [1], [9]—and write the O_2_ current, *I* (number of molecules transferred from air to blood per time), as
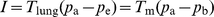
(1a)

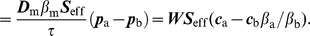
(1b)Here, the membrane includes the alveolar tissue barrier and the plasma (Fig. 12.7 in [1]). The driving force for the O_2_ transfer from air to erythrocyte is the partial pressure difference across the membrane and erythrocyte 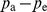, where *p*_a_ and *p*_e_ is the partial pressure of O_2_ in air adjacent to the membrane and in the erythrocyte, respectively, both averaged over the whole lung (Fig. 1). In Equation 1a, we express the current in terms of 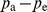 (total barrier: membrane and erythrocyte). Since the current across a series of resisters equals the current across any individual resister, we further express the current in terms of partial pressure difference across the membrane alone, 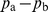. The inset of Fig. 1 shows the pressure profile across the membrane, with pressure *p*_b_ on the erythrocyte side of the membrane. The respective proportionality factors define the lung and membrane diffusing capacity, *T*_lung_ and *T*_m_ (number of molecules per time and pressure), used to determine the gas exchange capacity of the lung [1], [12]. Equation 1b expresses the membrane diffusing capacity in terms of 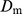, the diffusion coefficient of O_2_ in the membrane (area per time), the solubility 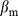 of O_2_ in the membrane (number of molecules per volume and pressure), the membrane thickness *τ* (including the thickness of alveolar tissue and the plasma, Table 1, [1], [2]) , and the surface area 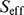 across which oxygen effectively diffuses (Fick's law; effective surface area is defined in Equation 4). To convert partial pressure difference to concentration difference, we used solubility *β*, which is the quantity of oxygen that becomes dissolved in a unit barrier volume if the partial pressure is raised by one unit, 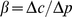 [1]. The quantity 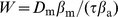 is the permeability of the membrane, defined as the number of O_2_ molecules crossing the membrane per unit surface area, unit time, and unit concentration difference between the two sides of the membrane, if both were air. Thus, the first part of Equation 1b describes the gas exchange as a three-phase system (air, membrane, erythrocyte), and the second part reduces the description to two phases, involving the permeability, which refers only to the membrane and air, and the rescaled concentration difference 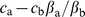, due to the fact that the medium on the far side of the membrane is not air, but erythrocyte.

Under steady-state conditions, the oxygen concentration 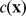, at position **x** in the diffusion space, i.e., acinar airways, obeys the stationary diffusion equation (Laplace equation) with appropriate boundary conditions:
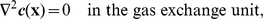
(2a)

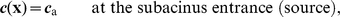
(2b)

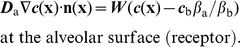
(2c)


The boundary condition 2b reflects that the gas exchanger, in which oxygen is transported by diffusion, is only a fraction of an acinus (Fig. 1), and that the air at the entrance has uniform oxygen concentration, 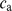. The boundary condition 2c states that the bulk diffusion flux in air in the direction normal to the surface, 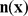, is equal to the transmembrane flux (current conservation). The condition accounts for screening, i.e., that the oxygen concentration at the alveolar surface, far away from the entrance, may be much smaller than at the entrance, 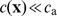, and highlights the importance of the ratio 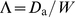, which has units of length. If this length is small, by virtue of the permeability being large, O_2_ is extracted quickly as it moves downstream from the entrance, and O_2_ visits only a small portion of the acinus before it crosses the membrane. Conversely, if this length is large, i.e., if the permeability is small, O_2_ travels a long distance until it crosses the membrane. Thus 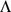 is the length of a typical diffusion path of an O_2_ molecule before it crosses the membrane, measured along the alveolar surface. Whence the term ‘exploration length’ for Λ. We treat *W*, and accordingly Λ, as a variable, and investigate the performance of the gas exchanger by analyzing the effective surface area and oxygen current as a function of *W* and Λ beyond the physiological range.

From the solution of the boundary-value problem, *c*(**x**), we compute the current as
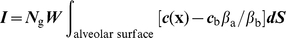
(3)

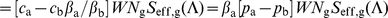
(4)where *S* is surface area; *N*_g_ is the number of gas exchangers in the lung; oxygen concentrations, solubilities, and partial pressures are as in Fig. 1; and Equation 4 expresses the integrated surface concentration, Equation 3, in terms of the concentration at the source, 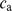, and the effective surface area of the gas exchanger, 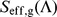, which is the area that would carry the same current in the absence of screening [4], [9], [13]. Equation 4 reduces the question of the oxygen current across the lung to the knowledge of the solubility of oxygen in air, partial pressure drop of oxygen across the membrane, number of gas exchangers, and effective surface area of a single gas exchanger. A renormalization treatment decomposes the surface into regions completely accessible and completely inaccessible to O_2_ molecules and calculates 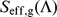 as area of the accessible region [4], [11], [13], [14]. For a membrane surface with fractal dimension 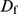 and source small compared to the gas exchanger, this is illustrated in Fig. 2 and leads to four regimes,
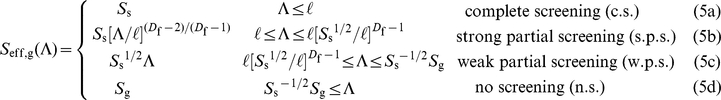
where 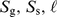 are the total surface area of the exchanger, cross-section area of the source, and alveolar side length. This gives 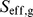 as a series of power laws of the exploration length, controlled by the fractal dimension, and identifies the relevant length scales downstream of 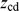.

The four regimes and associated length scales follow from the four geometric situations depicted in Fig. 2. In Equation 5a, the exploration length is less than the size of an alveolus, and only the region facing the source, with area *S*_s_ and depth less than an alveolus, contributes to the current. This is the case of complete screening. In Equations 5b and 5c, the exploration length is long enough that incoming molecules enter the hierarchy of small and large fjords of the fractal surface, but not long enough for the molecules to visit the entire surface. This is the partial screening regime. In Equation 5b the molecules visit a region one or several alveoli deep, but still shallow compared to the lateral size of the source, 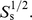 In Equation 5c, the depth of the visited region exceeds the lateral size of the source, and so does the lateral size of the visited region. In Equation 5d, the exploration length exceeds the perimeter of a planar cross section of the surface, and the molecules visit the entire surface before they cross the membrane. This is the case of no screening.

### Structural and Transport Parameters

Four structural parameters of the human lung are needed to calculate oxygen currents from Equations. 4 and 5: the alveolar side length, 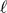; the cross-section area of the oxygen source, 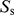; the surface area of the gas exchanger, 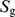; and the number of gas exchangers, 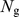. The values of 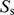, 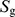 and 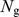 vary with *z*_cd_ (the values of *z*_cd_ as a function of the breathing rate are obtained below). We consider the gas exchanger as a cubic array of cubes of side length 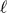 [9], [12], in which each cube contributes four of its six faces as surface for gas exchange. So 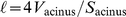, where *V*_acinus_ = 0.187 cm^3^ and *S*_acinus_ = 54 cm^2^ are the acinus volume and acinus surface area, respectively [12]. This gives 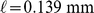. For the cross-section area of the source, we take 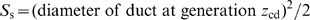 (square cross section), evaluation of which with data from [12] gives the values listed in Table 2. The cubic gas exchanger model is for convenience of calculation; a cylindrical/spherical model for the airways would give similar results. The surface area of the gas exchanger is obtained from 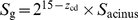, which reflects that the acinus comprises branching generations *z* = 15, …, 23 [12]. The resulting values are shown in Table 2. The number of gas exchangers is 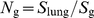, where 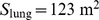 is the total surface area of the lung, taken as the arithmetic mean of the total alveolar surface area and total capillary surface area, following [2]. The value 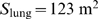 is obtained from data in [12], and the resulting values for *N*_g_ are listed in Table 2. The partial pressure differences across the membrane in Table 2 were obtained from 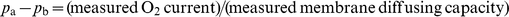, with experimental data discussed below. The membrane permeability in Table 1 is from Equation 1b and is evaluated using the listed values of diffusion coefficients, solubilities, and membrane thickness in Table 1. For the sake of comparison, we also list the values of diffusion coefficients and solubilities of O_2_, CO, and CO_2_ in water and air in Table 3.

**Table 2:** Structural and physiological data to compute O_2_ currents and compare computed and experimental values.

	Rest	Moderate exercise	Heavy exercise	Maximum exercise
Branching generation for convection-diffusion transition, 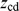	18	19	20	21
Gas exchanger	1/8 acinus	1/16 acinus	1/32 acinus	1/64 acinus
Side of alveolus, 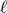 (cm)	0.0139	0.0139	0.0139	0.0139
Surface area of gas exchanger, 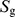 (cm^2^)	6.75	3.36	1.69	0.844
Cross-section area of source, 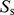 (cm^2^)	0.000800	0.000722	0.000648	0.000578
Number of gas exchangers, 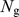	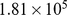	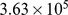	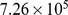	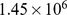
O_2_ partial pressure difference across membrane, 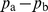 (Torr)	3.8 [2.8∼4]	7.9 [6.4∼10.3]	9.6 [7.2∼14.5]	8.7
Computed O_2_ current, *I* ( 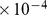 mol/s)	2.45 [1.8∼2.6]	9.69 [7.9∼12.6]	22.3 [16.7∼33.7]	38.2
Measured O_2_ current, *I* ( 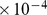 mol/s)	2.01	9.68	18.0	40.9
Computed membrane diffusing capacity, *T*_m_ 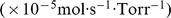	6.5	12.3	23.2	43.9
Measured membrane diffusing capacity, *T*_m_ 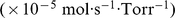	5.3 [5.0∼7.2]	12.3 [9.4∼15.2]	18.8 [12.3∼25.2]	46.8
Computed pulmonary efficiency, *η*	13%	26%	49%	94%
Measured pulmonary efficiency, *η*	11 [10∼5]%	26 [20∼32]%	40 [27∼53]%	100%

Levels of exercise are defined in the Methods section. Experimental uncertainties are reported as median [25 percentile∼75 percentile] (See Methods section and Text S1).

**Table 3:** The diffusion coefficients and solubilities of O_2_, CO, and CO_2_ in water and air at 300 K.

	Diffusion coefficient (cm^2^/s)		Solubility (mol⋅cm^–3^·Torr^–1^)
	water	air	water
O_2_	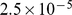	0.19	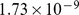
CO	-	0.19	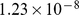
CO_2_	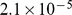	0.14	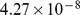

The solubilities of these gases in air can be calculated by idea gas law (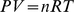).

### Breathing Regimes

The structural parameters, 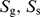, and 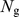 vary with the level of exercise because the branching generation 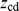, at which convection changes to diffusion, does so. Several authors [9], [12] proposed a concept—“acinus Peclet number” *P*_a_—to calculate 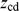. At branching generation *z*, the distance to travel to the end of the airway is of order 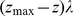, where *λ* is the mean length of an acinar duct and *z*_max_ is the total number of the branching generations (λ≈1 mm [12]; *z*_max_ = 23). So the mean diffusion velocity is 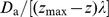, where 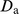 is the O_2_ diffusion coefficient in air. The acinus Peclet number is then defined as the ratio of convection velocity to diffusion velocity, 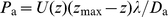, where 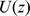 is the flow velocity at generation *z* (volume of air per unit time, divided by the cross-section area of the bronchial or acinar duct), which depends on the breathing rate. The convection-diffusion transition occurs when *P*_a_ = 1, and 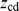 can be calculated (rounded to the nearest integer) from
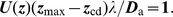
(6)Sapoval *et al.* [9], [12] analyzed the cases of rest and maximum exercise and obtained *z*_cd_ = 18, 21, respectively, for the two cases. To include intermediate breathing regimes, *z*_cd_ = 19, 20, and revisit the cases *z*_cd_ = 18, 21, we proceed as described in the next paragraph. This naturally leads to four distinct breathing regimes or levels of exercise, for which we assemble the experimental oxygen currents (for comparison with computed currents, Equations 4, 5) and membrane diffusing capacities (for partial pressure differences, 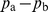). The discrete breathing regimes result from our focus on discrete gas exchange units— *z*_cd_ = 18, 19, 20, 21, leading to gas exchangers equal to 1/8, 1/16, 1/32, 1/64 of an acinus (dichotomous branching), The discrete screening regimes arise from geometric changes of the region incoming oxygen molecules visit before they cross the membrane.

The oxygen current, expressed as volume of O_2_ at STP per unit time (uptake rate), for a normal, healthy adult male at rest is about 270 ml/min [1], [12], [15]–[16]. The current at maximum exercise for well-trained athletes, which defines the maximum oxygen current a human lung can achieve, is about 5500 ml/min [17]. To obtain currents for the intermediate cases, *z*_cd_ = 19, 20, we calculated *U*(*z* = 19, 20) from Eq. 6, using 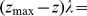
0.0363 cm (*z* = 19) and 0.028 cm (*z* = 20) (data from [12]. We then used mass conservation (equation of continuity at constant fluid density) to estimate the volumetric flow rate in the trachea for *z* = 19 and 20 [4]. For *z*_cd_ = 19, 20, the respective volumetric flow rates of air in the trachea are 30400 ml/min and 70760 ml/min, or less. At these two levels of volumetric flow rates, there are two studies reporting oxygen currents and diffusing capacities. For heavy exercise, Weibel (Table 10.2 in [1]) reported volumetric flow rates in the trachea, 68100 ml/min, which is very close to 70760 ml/min. So we associate heavy exercise with *z*_cd_ = 20. The corresponding oxygen current at this level is 2420 ml/min, and the diffusing capacity for oxygen of the whole lung is about 100 ml/min/Torr. For 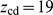 (“moderate exercise”), we take the linear regression relation between oxygen current and volumetric flow rates in the trachea reported by Newstead [18] to calculate the oxygen current, and found it to be 1360 ml/min when the volumetric flow rate is 30400 ml/min. Borland et al. [19] measured the membrane diffusing capacity att an oxygen current of 1300 ml/min, which is very close to 1360 ml/min. We therefore associate Borland's oxygen current and membrane diffusing capacity with *z*_cd_ = 19. Conversion of currents into mol/s, conversion of lung diffusing capacities into membrane diffusing capacities, and averaging of multiple measurements, gives the values reported in Table 2 for *z*_cd_ = 18, 19, 20, 21.

## Results/Discussion

### Fault Tolerance

Figure 2 displays the screening regimes, length scales, and O_2_ current for a single gas exchanger at rest. It shows that only for 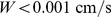 is the surface unscreened and the effective surface area equal to the total surface area of the exchanger, 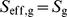; for all larger permeabilities, we have 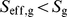. The most striking feature is the extended horizontal plateau. If *W* increases beyond 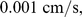 the effective surface area drops, and the drop exactly cancels the increase in *W*, Equations 5c, leading to a constant current over more than three decades of *W*. The constant current provides protection against loss of permeability under environmentally adverse or disease conditions. The current remains stable even if the permeability drops from its normal value, 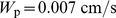, by a factor of seven. We refer to this robustness, 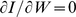, as maximum fault tolerance. It is similar to a “constant-current source” in an electric circuit, designed to deliver a constant current under variable load (here, surface resistance). In the lung, it autonomously results from the hierarchical tree structure, without any feedback loop, and has not been observed before. Horizontal plateaus have been noted in other branched structures [20].

### Transformation of 180,000 Gas Exchangers into 1,500,000 Gas Exchangers

We computed O_2_ currents at four levels of exercise (Table 2). They are compared with experimental currents and other benchmarks in Figs. 3 and 4. A rich spectrum of results and concepts emerge—number of gas exchangers, current across each exchanger, effective surface area in each exchanger, pressure difference 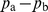
—to monitor how the lung ramps up the current:

Computed and experimental currents agree within a factor of 0.9–1.2 (Fig. 3A). To the best of our knowledge, this is the first time currents are computed from first principles. The agreement is remarkable because the computed currents come from a model of gas exchange with minimum number of structural parameters 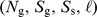 and transport parameters 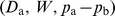. The model requires no data on branching (number/width/length of daughter ducts), shape of alveoli, or membrane epithelia, and no computation of concentration maps, 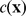, in the airways.

Increased ventilation under increasing exercise pushes the transition from convection to diffusion, 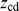, to more distant branchings [9], which reduces the size of gas exchangers, 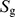, but increases their number, 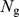. The current across an individual gas exchanger remains approximately constant, 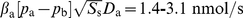, and so does the effective surface area, 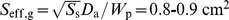 (Equations. 4, 5c, Table 1 and 2). Thus, increased ventilation transforms 180,000 exchangers with 1.4 nmol/s per exchanger into 1,500,000 exchangers with 2.6 nmol/s per exchanger. This shifts the plateaus in Fig. 4 upward and increases the current, mostly by the increase in 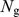. It shows that the current increase occurs under strongly variable conditions in the bronchial airways (convective air flow) and capillaries (blood flow), but only weakly varying conditions in individual gas exchangers, making the acinar airways a self-regulated system, largely decoupled from the dynamics of the input and output system.

A figure of merit stripped of the dependence on 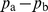 is the pulmonary efficiency, defined as the ratio of effective to total surface area, Equation 7a, and measured as the ratio of experimental membrane diffusing capacity, 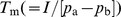, to morphometric diffusing capacity, 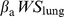 [1], [2], [12], Equation 4c:
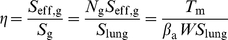
(7a, b, c)where 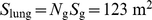 is the total surface area of the lung (Table 2). Our efficiencies from Eq. 7a are 13%, 26%, 49%, and 94% at rest and moderate/heavy/maximum exercise; the experimental values, from Equation 7c, are 11%, 26%, 40%, and 100%. The agreement is excellent (Fig. 3B). The values also agree well with earlier calculations, 10–40% at rest, and 100% at maximum exercise [4], [9], [12], [20]–[22]. The efficiency increases with increasing exercise because increased ventilation reduces screening—according to Eq. 7a by the decrease in 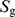 (1/8, …, 1/64 acinus); according to Eq. 7b by the increase in 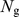
—both at constant 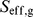 and constant lung volume. The tenfold increase in efficiency allows the lung to increase the O_2_ current by a factor of 20 with only a twofold increase in pressure difference across the membrane 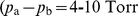, Table 2). So the area screened at low efficiency acts as “spare area” and is the principal source of the current increase. It transforms 180,000 screened gas exchangers with 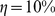 per exchanger into 1,500,000 unscreened exchangers with 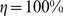 per exchanger.

### Adaptation to Changing Resources and Demands, Optimum Performance

In Fig. 4, which plots the oxygen current as a function of membrane permeability for all four levels of exercise, we now focus on currents at fixed permeability and variable exercise. The line 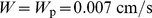 intersects all four plateaus and runs almost through the “knee point” at maximum exercise. This optimizes switching from rest to exercise in extraordinary ways: If the lung operated in region C, it would have maximum fault tolerance over a maximum interval of permeabilities at maximum exercise, but waste more than 99% of its surface area due to massive screening (Equations 5b, 5c, 7a). If the lung operated in region A, no waste would occur, but all fault tolerance would be lost and increased ventilation would not increase the current. Only in region B are four major engineering targets met—maximum current at minimum permeability (knee point at maximum exercise); maximum current increase from rest to exercise (maximum response to increased ventilation); no waste of resources (surface area) at maximum exercise; and maximum fault tolerance over a broad interval of lower than normal permeabilities at rest (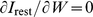 for 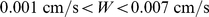). This should be contrasted with the expectation that a maximum current would require maximum permeability, all other parameters held constant; that a large current increase might require a large increase in partial pressure difference across the membrane or an increase in total surface area of the lung; that a maximum current might require overdesign of resources; or that a drop in permeability would inevitably lead to a drop in current.

The location of 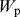 near the knee point identifies a second notable property: far to the right of the knee, diffusing O_2_ molecules explore only a small part of the membrane surface (“underexploration,” short residence time of O_2_ in the gas exchanger); far to the left, molecules explore the surface multiple times before crossing the membrane (“overexploration,” long residence time); at the knee point, molecules explore the surface essentially once. Thus the knee, at maximum exercise, is the unique point at which O_2_ molecules visit the entire surface (maximum exploration) at minimum residence time. This optimizes transport at the level of microscopic dynamics. If, alternatively, the knee were further to the right (as it would if 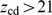), the lung could generate a current larger than 4 mmol/s if 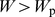. The fact that nature has not selected this option suggests that it is more important to keep the permeability low, to maintain a strong barrier against intruders and fluid effusion from capillaries [18], than to generate larger currents.

### Alternative Gas-Exchanger Architectures and Models

**Figure 5 pcbi-1000902-g005:**
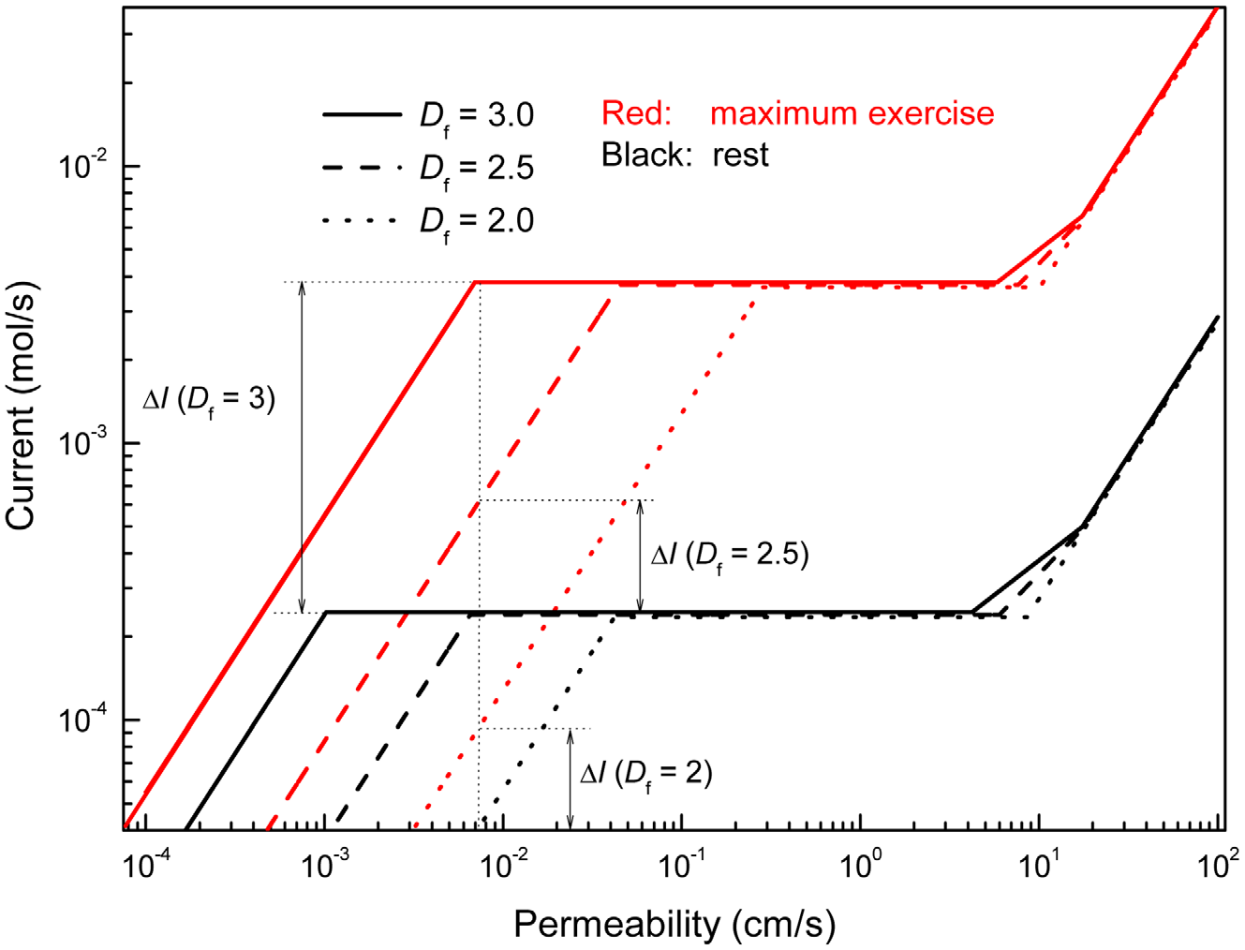
Computed oxygen currents for alternative architectures. Currents as a function of *W* are computed from Eqs. 4 and 5 for different fractal dimensions, *D*_f_, at fixed 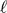 and side length of the gas exchanger (side length of 1/64 acinus, column 5 of Table 2). Other transport parameters in the equations are from Table 1.

In Fig. 5, we ask what if the gas exchangers were less than space-filling, 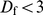. At 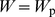, as the fractal dimension drops from 3.0 to 2.5 to 2.0 (flat surface), the current at maximum exercise would drop from 4 mmol/s to 0.6 mmol/s to 0.09 mmol/s; the current increase, from rest to exercise, would drop from 20- to 3- to 2-fold and all fault tolerance would be lost. Thus none of the achievements in region B of Fig. 4 could be realized with 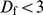. E.g., to achieve a current comparable to 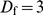, but with a 2.5-dimensional surface, would require a membrane permeability of 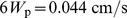. Decreasing *D*_f_ shifts the curves to the right and requires increasing permeabilities to achieve currents identical those at *D*_f_ = 3. The reason is that, at fixed side length of the gas exchanger, the surface area of the exchanger drops with decreasing *D*_f_ so that a large *W* has to compensate for a small *S*_g_ (Equations 5c, d).

**Figure 6:**
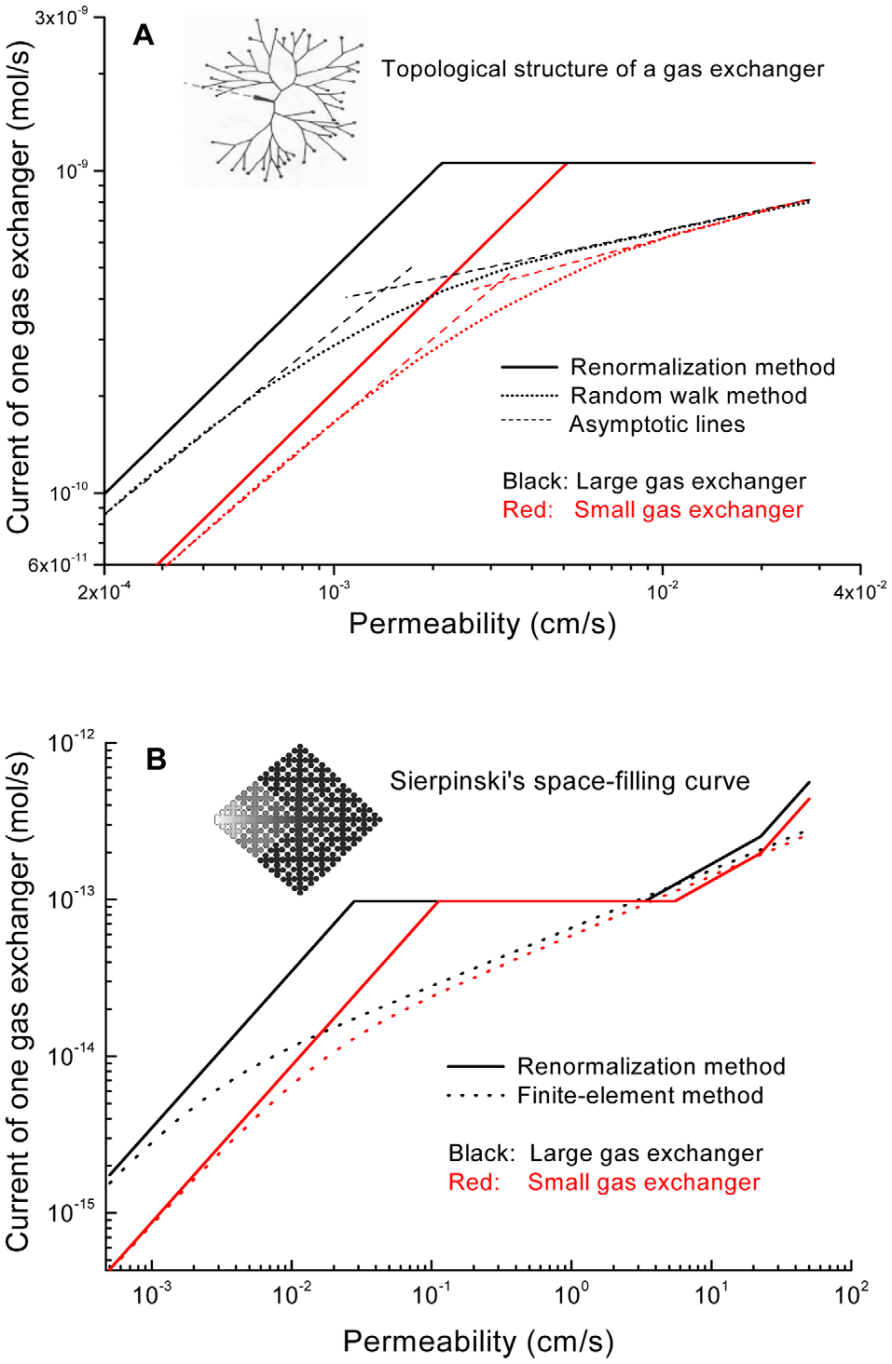
O_2_ currents, at rest, across other gas-exchanger models. (A) Random-walk computations for largest and smallest specimen of eight 1/8 human acini, modeled as network of acinar ducts [21], [22]. (B) Finite-element computations for Sierpinski's plane-filling curves [32], as planar models of a 1/8 and 1/128 acinus (*D*_f_ = 2, [4]). Both panels also show the respective currents from the renormalization method. Inset in (B): concentration field, (*c*(x)−*c*_b_ *β*_a_/*β*,)/(*c*_a_−*c*_b_ *β*_a_/*β*,), in the 1/8 acinus (column 2 of Table 2).

In Fig. 6, we compare the renormalization method (RM), Equation 5, with earlier computations —(A) for three-dimensional models of a 1/8 acinus, in which the diffusion-reaction problem, Equation 3, was approximated by random walks (RW) with absorbing boundary conditions [21], [22]; and (B) for two-dimensional models of a 1/8 and 1/128 acinus, in which Equation 3 was solved by the finite-element method (FEM; [4], Text S1). The RM, RW, and FEM currents agree within a factor of order one over 5 orders of magnitude of the permeability and nearly 3 orders of magnitude of the current. This is excellent agreement considering that the RW and FEM treatments trace out every duct detail (Fig. 6), while the RM evaluates Equation 3 with 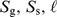 as sole structural input (see also [13], [23]). The sharp transitions in the RM currents, which result from the decomposition of the surface into regions completely accessible/inaccessible to O_2_ molecules (Fig. 2), are smooth in the RW and FEM treatment: e.g., the horizontal plateau is transformed into a weakly *W*-dependent current. Thus the RM pinpoints changes in screening not easily detectable in purely numerical computations—similar to that, in adsorption of gases on solids, different models may or may not produce a knee and plateau in the adsorption isotherm [24]. In Fig. 6, the knee marks the transition from overexploration to underexploration of the membrane surface and yields the cross-section area of the source; in adsorption the knee marks the transition from submonolayer to multilayer coverage and yields the surface area of the solid.

The RM, RW, and FEM currents also agree, within a factor of order one, in terms of the knee points on the *W* axis. The RW currents for the two different realizations of a 1/8 acinus, but with identical source, merge at large *W*, in agreement with the RM prediction that screened currents depend only on the cross-section area of the source, Equation 5a–c. The RW currents at 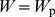 are 0.5–0.6 nmol/s, which is about half of the experimental value at rest, 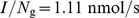 (Table 2), because the 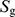
's in [21]–[22] are smaller than 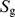 in Table 2, which is averaged over many 1/8 acini.

This comparison of the RM, RW, and FEM currents provides important validation of the RM treatment and fractal model. Are there uncertainties of the model outside the diffusion-reaction framework, Equations 2–4? The results in Figs. 2–6 are based on a constant lung volume, 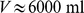. But breathing is dynamic and the volume of the lung changes periodically. If the fractional volume change is 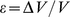, then the fractional linear change, i.e., the side length of alveoli and acini, will be *ε*/3. At rest, the tidal volume for a normal adult male amounts to a volume change of 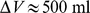 [1], whence 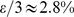. Evaluating Equation 4, 5, 7 with accordingly changed values of the structural parameters, we find that the fractional change in the oxygen current is 2.9%, and the pulmonary efficiency changes from 13.8% to 13.4%. We consider both changes insignificant. During maximum exercise, from functional residual capacity to peak inspiration, the lung volume changes as much as 50% [1]. In this case, the oxygen current changes by 17%, and the pulmonary efficiency increases from 94% to 100%.

During the periodic expansion and contraction of the lung, the air flow and gas mixing in the acinar airways can be chaotic [25], [26]. The chaotic patterns provide the time-resolved fluid-dynamic details of what happens at the transition between convection and diffusion. Upstream of the transition, velocity vectors point mostly downstream, along the duct axis; downstream of the transition, velocity vectors point mostly transverse to the duct axis (diffusion to duct wall). In the transition region, some velocity vectors point along the duct axis, others transverse to the duct axis, giving rise to eddy-like instantaneous flow patterns. In the stationary diffusion-reaction framework, Equations 2, 3, these flow patterns are averaged out, and the average yields the boundary condition at the source, Equation 2b. One may view the flow patterns as fluctuations around the stationary average and examine their effect on the gas exchange. From the perspective of length scales, the effect is minimal: a typical eddy has a diameter of 0.005–0.01 cm [25], [26], which is small compared to the side length of the source (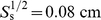 for *z*_cd_ = 21; 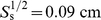 for *z*_cd_ = 18), side length of the gas exchangers (0.36 cm for *z*_cd_ = 21; 0.57 cm for *z*_cd_ = 18), and exploration length, 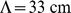. From the perspective of the current, the fluctuations have no effect: in the mean-field description provided by the diffusion equation, Equation 2, they give the oxygen source, which is a dividing surface of zero thickness in Equation 2b, a nonzero thickness of the order of the eddy size, centered at the subacinus entrance. But the oxygen concentration is identical on both sides of the dividing surface, so the fluctuations are less than *c*_a_ as often as they are larger, and the stationary oxygen current across the lung is the same as in the absence of fluctuations. In terms of dynamics, the eddies create a well-stirred chemical reactor, the prerequisite for a source with uniform time-averaged oxygen concentration [4].

The branching pattern of the fractal model in our study is symmetric, in line with that branching in the acinar airways is symmetric to a significant degree [12]. But the bronchial tree is asymmetric [27], [28], and the question arises how asymmetry would influence our calculations. Asymmetry is important in the bronchial tree (convection, low *z*) where the flow distribution depends sensitively on the aspect ratio of the daughter branches [6], [29]. Asymmetry is not important in the acinar tree (diffusion, high *z*) because diffusion currents are driven by local concentration gradients, which depend predominantly on the distance to the nearest wall, and only little on geometric factors like width, length, and angle of daughter branches. Insensitivity of diffusion to asymmetry is supported by the RW results in [21], [22], which include various asymmetries, but depend mostly on the size of the subacinus (Fig. 6A). However, little is known whether an asymmetric flow distribution in the bronchial tree can propagate all the way to the acinar tree and generate significantly different oxygen concentrations at the entrances of different gas exchangers. If so, then *c*_a_ in Equations 1b, 2b, 4 should be an appropriately averaged entrance concentration.

### Conclusions

We have shown that oxygen exchange across the alveolar membrane can be successfully modeled as diffusion-reaction process bounded by a fractal, space-filling surface; that the fractal nature of the surface is key to the high performance of the gas exchanger; and that the operation of the system can be understood in terms of variable degrees of screening under different physiological conditions.

The results in Fig. 3 (validation of the model) and Fig. 4 (prediction of currents at arbitrary permeabilities) were calculated by plugging seven numbers (Rows 1&6 in Table 1; Rows 3–7 in Table 2) into Equations 4, 5, which requires no more than a pocket calculator. The success of the calculation in Fig. 3 demonstrates the power of the fractal model and associated physical insight—transformation of 180,000 screened gas exchangers into 1,500,000 unscreened gas exchangers, accompanied by an increase of the oxygen current by a factor of 20, essentially at constant lung volume, surface area, partial pressure difference across the membrane, and membrane permeability. The success of the calculation in Fig. 4 provides a robust map of the vast territory of membrane permeabilities different from the normal physiological value. The success of the “pocket-calculator formula,” Equation 4, 5, promises a robust map of respiratory performance of the lung in other species [1], [2], [8].

We demonstrated that the space-filling architecture provides optimum adaptation to changing demands—the ability to switch from a low oxygen current at rest to high currents at exercise (vertical transition in Fig. 4), self-regulated by diffusional screening, without external control circuits. Such adaptation is one hallmark of robustness in systems biology [30]. At the same time, the architecture provides optimum adaptation to changing resources—maintenance of a constant oxygen current in the event of a change in permeability, surface area, or other operational parameters (homeostasis; horizontal transition in Fig. 4), again without external controls. Such changes may occur in pulmonary edema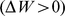, inhalation of aerosols 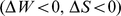, poor ventilation in asthma 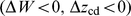, pneumonia 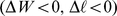, emphysema 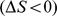, lung surgery 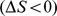, hyperbaric oxygen treatment 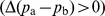. Insensitivity to specific operational parameters is the second hallmark of robustness in biological systems. While such insensitivity is of outstanding value for stable oxygen delivery under less than perfect conditions, it may make direct experimental tests or therapeutic applications, in which departures from normal oxygen delivery are observed, feasible only under severe departures from normality. Necessary for such tests and applications will be quantitative estimates of Δ*W*, Δ*S*, Δ*z*_cd_, 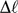, and 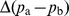 under various disease and treatment conditions. To the best of our knowledge, such estimates have yet to be developed.

We reverse-engineered the lung's performance characteristics by monitoring how the oxygen current varies as we vary transport and structure parameters, here *W* and *D*_f_, over values far from those found in the lung, similarly to how reverse engineering of biomolecules requires experiments at temperatures far from ambient temperature [31]. The resulting understanding of how structure determines function, how a single three-dimensional surface can create a platform for coexistence of multiply optimized properties, gives new meaning to the statement that “Lebesgue-Osgood monsters are the very substance of our flesh” ([3], p. 149, 159).

## Supporting Information

Text S1 Description of physiological data and model computations. Section 1: Experimental values of membrane diffusion capacities at four levels of exercises. Section 2: Oxygen currents across single gas exchangers–random-walk computations and finite-element computations.
(0.19 MB DOC)
