# Supplementary material for: Reverse Engineering of Oxygen Transport in the Lung: Adaptation to Changing Demands and Resources through Space-Filling Networks
Source: PLoS Comput Biol. 2010 Aug 26;6(8):e1000902. doi: 10.1371/journal.pcbi.1000902 (PMC2928740; doi:10.1371/journal.pcbi.1000902)
Supplement: Text S1 — Description of physiological data and model computations. Section 1: Experimental values of membrane diffusion capacities at four levels of exercises. Section 2: Oxygen currents across single gas exchangers–random-walk computations and finite-element computations. (0.19 MB DOC) [file pcbi.1000902.s001.doc]

Hou, Gheorghiu, Huxley, Pfeifer

**Supporting Text S1**

**Description of physiological data and model computations**

**1. Experimental values of membrane diffusion capacities at four levels of exercises**

**(1) At rest.** Many studies have measured the diffusion capacity for carbon monoxide of the alveolar membrane (*T*_m, CO_) or the whole lung (*T*_L,CO_) at rest (Table 1). Because of the difference in solubility and diffusivity of oxygen and carbon monoxide molecules in water, the diffusion of oxygen is 1.23 times faster than carbon monoxide, so $T_{\text{m, O}_{2}}\sim1.23T_{\text{m, CO}}$ (see e.g. Ref. [S1]). Some studies only measured *T*_L, CO_. In those cases, we take the classic model of pulmonary diffusion proposed by Roughton and Forster [S2], which describes the two-resistor model for CO transfer, to calculate *T*_m, CO_:

$1/T_{\text{L, CO}}=1/T_{\text{m, CO}}+1/(\theta_{\text{CO}}V_{\text{c}})$ (S1)

where $\theta_{\text{CO}}$ is the rate of carbon monoxide uptake by whole blood and combination with hemoglobin, and $V_{\text{c}}$is the pulmonary capillary blood volume.

We take the median value $T_{\text{m, O}_{2}}=71.3$ $\text{ml/min/Torr}$ for further calculation (Table 1). With equation $I=T_{\text{m}}(p_{\text{a}}-p_{\text{b}})$, and empirical value of oxygen current, *I* = 270ml/min (Table 1 in main text), we have $p_{\text{a}}-p_{\text{b}}=270\text{ ml/min}/71.3\text{ ml/min/Torr}=3.8\text{ Torr}$.

**Table 1.** Comparison of values of membrane and whole lung diffusion capacity for oxygen and carbon monoxide from 17 studies.

| Reference | (ml/min/Torr) | (ml/min/Torr) | (ml/min/Torr) | $V_{\text{c}}$ (ml) |
| --- | --- | --- | --- | --- |
| Lewis et al. (1958) [S3] | 120.5 | 98 | 29 | 65 |
| Bedell et al. (1962) [S4] | 64.9 | 52.8 | 29 | - |
| Roughton (1964) [S5] | 68.9 | 56 | 33 | 79 |
| Johnson et al. (1965) [S6] | 68.9 | 56 | 30 | 75 |
| Krumholz (1966) [S7] | 131.6 | 107 | 39 | 99 |
| Hyde et al. (1967) [S8] | 75 | 61 | 44 | 104 |
| Cross et al. (1973) [S9] | 89.2 | 72.5 | 45 | 121 |
| Crapo et al. (1982) [S10] | 70.1 | 57 | 29 | 87 |
| Guenard et al. (1987) [S11] | 97.2 | 79 | 30 | 78 |
| Borland et al. (1989) [S12] | 88.4 | 71.9 | 40 | 90~115 |
| Huang et al. (1994) [S13] | 71.3 | 58 | 30.5 | - |
| Hsia et al. (1995) [S14] | 63.6 | 51.7 | 32 | 108 |
| Puri et al. (1995) [S15] | 73.4 | 59.7 | 30.1 | - |
| Zanen et al. (2001) [S16] | 67.8 | 55.1 | 28.8 | 86.3 |
| Tamhane et al. (2001) [S17] | 57.7 | 46.9 | 29.3 | 110 |
| Huang et al. (2002) [S18] | 69.6 | 56.6 | 30.1 | - |
| Zavorsky et al. (2004) [S19] | 131.2 | 106.7 | 46.2 | 116.3 |
| Median | 71.3 | 58 | 30.1 | 99 |
| 25 percentile | 68.9 | 56 | 29.3 | 79 |
| 75 percentile | 89.2 | 72.5 | 39 | 108 |

**(2) Moderate exercise.** Borland et al. (2001) [S20] measured steady-state membrane diffusion capacity of nitric oxide (NO) and carbon monoxide (CO) on moderate exercise in men [S20]. The oxygen uptake rate at this level of exercise was reported to be 1300 ml/min. Based on two different assumptions of the reaction rate of NO with hemoglobin, the authors gave an upper and lower limit for membrane diffusion capacity of CO, , and therefore two limits of (126.7 ~ 204.7 ml/min/Torr). We take the median value of them 165.7 ml/min/Torr as the experimental value at this level of exercise.

**(3) Heavy exercise.** At this level of exercise, Weibel (1984) reported that the oxygen current is 2420 ml/min, and the corresponding diffusion capacity of the whole lung, is 100 ml/min/Torr [S1]. We take the Roughton-Forster equation [S2, S9] to calculate the membrane diffusion capacity, , where $\theta_{\text{O}_{2}}$is the reaction rate of oxygen and hemoglobin and $V_{\text{c}}$ is the capillary blood volume at this level of exercise. $V_{\text{c}}$ is unknown. But, since recruitment of pulmonary capillaries increases with exercise intensity [S21], $V_{\text{c}}$ must be between the values at moderate exercise, 127 ml [S20], and the morphometric value, which is also the maximum, 194 ml [S22]. So, we take the middle value, 160.5 ml, to do the calculation. It is difficult to measure $\theta_{\text{O}_{2}}$ [S1]. Weibel presented the value of it in desaturated blood (1.5ml/ml/min/Torr) and the effective mean value of it (0.85ml/ml/min/Torr). We take these two values to calculate the upper and lower limit of at this level of exercise: $T_{\text{m, O}_{2}}=(171\sim374.5)\text{ ml/min/Torr}$. The mean value is 272.7 ml/min/Torr.

**(4) Maximum exercise.** We take the value of oxygen current at maximum exercise for well-trained athletes, 5500 ml/min, because it is the maximum value the human lung can reach. We assume that at this level of exercise, the spare surface area, which play roles of spare resource at lower level of exercises and rest, is fully used. Therefore, screening disappears at maximum exercise. This assumption is supported by two-dimensional numerical simulation in a Hilbert acinus [S22-24]. The pulmonary efficiency is 100%, and the membrane diffusion capacity is equal to the morphometric value of it $T_{\text{m, morph}}=W\beta_{\text{a}}N_{\text{g}}S_{\text{g}}$.

**2. Oxygen currents across single gas exchangers: random-walk computations and finite-element computations**

The currents from the random-walk method in Fig. 6A, for the two specimens of a gas exchanger analyzed by Felici *et al.* [S25-26], were calculated from $I=\beta_{\text{a}}(p_{\text{a}}-p_{\text{b}})W\eta S_{\text{g}}$, where *η* and *S*_g_ are the pulmonary efficiency and gas exchanger surface area, respectively, reported in Fig. 5 in Ref. [S25] for the largest and smallest gas exchanger, and $p_{\text{a}}-p_{\text{b}}=3.8\text{ Torr}$ at rest (Table 1 in main text). The corresponding currents from the renormalization method (RM) were calculated from $I=\beta_{\text{a}}(p_{\text{a}}-p_{\text{b}})WS_{\text{eff,g}}(\Lambda)$ and Equation 3, with *S*_g_ from Fig. 5 in Ref. [S25], $S_{\text{s}}=0.000800\text{ cm}^{2}$ at rest (Table 1 in Main text), and $D_{\text{a}}=0.19\text{ cm}^{2}\text{/s}$ [S25-26].

The currents from the finite-element method were obtained by solving the boundary-value problem Equation 2 in two dimensions (2D) with the following data:

$\left.\begin{matrix} (S_{\text{g}})_{\text{2D}}=8.69\text{ cm} \\ (S_{\text{s}})_{\text{2D}}=0.0283\text{ cm} \end{matrix}\right\}\text{ (Sierpinski model for gas exchanger at rest)}$, (S2a)

$\left.\begin{matrix} (S_{\text{g}})_{\text{2D}}=2.17\text{ cm} \\ (S_{\text{s}})_{\text{2D}}=0.0219\text{ cm} \end{matrix}\right\}\text{ (Sierpinski model for gas exchanger at max exercise)}$, (S2b)

, (S2c)

$D_{\text{a}}=0.243\text{ cm/s}$, S2d)

$W_{\text{p}}=0.00739\text{ cm/s}$, (S2e)

where the values in Equations S2a,b are from Ref. [24], and Equations S2c-e are from Table 1 in main text, observing that the alveolar side length, diffusion coefficient, and permeability have values and units independent of the dimensionality of the diffusion space. The concentration difference in two dimensions, $(c_{\text{a}}-c_{\text{b}}\beta_{\text{a}}/\beta_{\text{b}})_{\text{2D}}$, was calculated from that in three dimensions, $(c_{\text{a}}-c_{\text{b}}\beta_{\text{a}}/\beta_{\text{b}})_{\text{3D}}=\beta_{\text{a}}[p_{\text{a}}-p_{\text{b}}]$ (Equation 4**)** and Table 1 in main text, as follows. If *N* molecules occupy a volume *V*, then one molecule occupies a cube of side length $(V/N)^{1/3},$ basal area $(V/N)^{2/3},$ and volume $V/N.$ This gives

$$(c_{\text{a}}-c_{\text{b}}\beta_{\text{a}}/\beta_{\text{b}})_{\text{2D}}$$

$$=[(c_{\text{a}}-c_{\text{b}}\beta_{\text{a}}/\beta_{\text{b}})_{\text{3D}}]^{2/3}$$

$$=[(0.197\times10^{-6}\text{ mol/cm}^{3})\cdot(6.02\times10^{23}\text{ particles}\cdot\text{mol}^{-1})]^{2/3}$$

$=2.42\times10^{11}\text{ particles/cm}^{2}=4.02\times10^{-13}\text{ mol/cm}^{2}$. (S2f)

for O_2_ transport at rest. We note that the formula $(c_{\text{a}}-c_{\text{b}}\beta_{\text{a}}/\beta_{\text{b}})_{\text{2D}}$ $=[(c_{\text{a}}-c_{\text{b}}\beta_{\text{a}}/\beta_{\text{b}})_{\text{3D}}]^{2/3}$ requires concentrations to be in units of number of particles per unit volume and number of particles per unit area, respectively, for purely dimensional reasons. If the 3D concentration were in moles per volume, the right hand side of $(c_{\text{a}}-c_{\text{b}}\beta_{\text{a}}/\beta_{\text{b}})_{\text{2D}}$ $=[(c_{\text{a}}-c_{\text{b}}\beta_{\text{a}}/\beta_{\text{b}})_{\text{3D}}]^{2/3}$ would have units of mol^2/3^⋅cm^–2^ instead of $\text{cm}^{-2}$.

The currents from the renormalization method for the models in two dimensions were computed from

$I_{2\text{D}}=(c_{\text{a}}-c_{\text{b}}\beta_{\text{a}}/\beta_{\text{b}})_{\text{2D}}\cdot W\cdot(S_{\text{eff,g}}(\Lambda))_{\text{2D}}$, (S3)

with $D_{\text{f}}=2$ (plane-filling membrane) and S52a-f. Equation S4 is the renormalization result in two dimensions, for a membrane with fractal dimension *D*_f_, $1\leq D_{\text{f}}\leq2,$ from Ref. [24].

It follows that, in two dimensions, the current in the renormalization method is controlled by the size of the source, (*S*_s_)_2D_, in the strong partial and complete screening regime, Equations S4a, b, but not for weak partial screening, Equation S4c. So the renormalization currents at large *W* in Fig. 6B differ for the large and small Sierpinski exchanger because the size of the source is different for the two exchangers, Equations S4a, b, but the currents coincide in the plateau regime.

**Supporting References**

1. Weibel ER (1984) The Pathway for Oxygen. Cambridge, USA: Harvard University Press.

2. Roughton FJW, Forster RE (1957) Relative importance of diffusion and chemical reaction rates in determining rate of exchange of gases in the human lung, with special reference to true diffusing capacity of pulmonary membrane and volume of blood in the lung capillaries. J Appl Physiol 11:290-302.

3. Lewis BM, Lin TH, Noe FE, Komisaruk R (1958) Measurement of pulmonary capillary blood volume and pulmonary membrane diffusing capacity in normal subjects: Effects of exercise and position. J Clin Invest 37:1061-1070.

4. Bedell GN, Adams RW (1962) Pulmonary diffusing Capacity during rest and exercise: a study of normal persons and persons with atrial septal defect, pregnancy, and pulmonary disease. J Clin Invest 4:1908-&.

5. Roughton FJW (1964) Section3. Respiration. Handbook of Physiology, ed Fishman AP. Bethesda, MD: The American Physiological Society, Vol I.

6. Johnson RL, Taylor HF, Lawson WH (1965) Maximal diffusing capacity of lung for carbon monoxide. J Clin Invest 44:349-&.

7. Krumholz RA (1966) Pulmonary membrane diffusing capacity and pulmonary capillary blood volume: an appraisal of their clinical usefulness. Am Rev Respir Dis 94(2):195-200.

8. Hyde RW, Rynes R, Power GG, Nairn J, Moyer P (1967) Determination of distribution of diffusing capacity in relation to blood flow in human lung. J Clin Invest 46:463-&.

9. Cross CE, Gong H, Kurpersh.Cj, Gillespi.Jr, Hyde RW (1973) Alterations in distribution of blood-flow to lungs diffusion surfaces during exercise. J Clin Invest 52:414-421.

10. Crapo R, Morris A, Gardner R (1982) Reference values for pulmonary tissue volume, membrane diffusing capacity, and pulmonary capillary blood volume. *Bull. Europ. Physiopah. Resp.* 18:893-899.

11. Guenard H, Varene N, Vaida P (1987) Determination of lung capillary blood-volume and membrane diffusing: capacity in man by the measurements of NO and CO Transfer. Respir Physiol 70:113-120.

12. Borland CDR, Higenbottam TW (1989) A simultaneous single breath measurement of pulmonary diffusing: capacity with nitric-oxide and carbon-monoxide. Eur Respir J 2:56-63.

13. Huang YCT, Helms MJ, Macintyre NR (1994) Normal values for single exhalation diffusing: capacity and pulmonary capillary blood-flow in sitting, supine positions, and during mild exercise. Chest 105:501-508.

14. Hsia CCW, McBrayer DG, Ramanathan M (1995) Reference values of pulmonary diffusing: capacity during exercise by a rebreathing technique. Am J Respir Crit Care 152:658-665.

15. Puri S et al*.* (1995) Reduced alveolar capillary membrane diffusing-capacity in chronic heart-failure: Its pathophysiological relevance and relationship to exercise performance. Circulation 91:2769-2774.

16. Zanen P, van der Lee I, van der Mark T, van den Bosch JMM (2001) Reference values for alveolar membrane diffusion capacity and pulmonary capillary blood volume. Eur Respir J 18:764-769.

17. Tamhane RM, Johnson RL, Hsia CCW (2001) Pulmonary membrane diffusing capacity and capillary blood volume measured during exercise from nitric oxide uptake. Chest 120:1850-1856.

18. Huang YCT, O'Brien SR, MacIntyre NR (2002) Intrabreath diffusing capacity of the lung in healthy individuals at rest and during exercise. Chest 122:177-185.

19. Zavorsky GS, Quiron KB, Massarelli PS, Lands LC (2004) The relationship between single-breath diffusion capacity of the lung for nitric oxide and carbon monoxide during various exercise intensities. Chest 125:1019-1027.

20. Borland C, Mist B, Zammit M, Vuylsteke A (2001) Steady-state measurement of NO and CO lung diffusing capacity on moderate exercise in men. J Appl Physiol 90:538-544.

21. Hsia CCW (2002) Recruitment of lung diffusing capacity - Update of concept and application. Chest 122:1774-1783.

22. Weibel ER, Sapoval B, Filoche M (2005) Design of peripheral airways for efficient gas exchange. Respir Physiol Neurobiol 148:3-21.

23. Sapoval B, Filoche M, Weibel ER (2002) Smaller is better-but not too small: A physical scale for the design of the mammalian pulmonary acinus. Proc Natl Acad Sci USA 99:10411-10416.

24. Hou C, Gheorghiu S, Coppens M-O, Huxley VH, Pfeifer P (2005) Gas diffusion through the fractal landscape of the lung: How deep does oxygen enter the alveolar system? Fractals in Biology and Medicine, eds Losa GA, Merlini D, Nonnenmacher TF, Weibel ER, Basal: Birkhäuser, Vol IV, pp 17-30.

25. Felici M, Filoche M, Sapoval B (2004) Renormalized random walk study of oxygen absorption in the human lung. Phys Rev Lett 92:068101(1-4).

26. Felici M, Filoche M, Straus C, Similowski T, Sapoval B (2005) Diffusional screening in real 3D human acini - a theoretical study. Respir Physiol Neurobiol 145:279-293.
